# Correction to: Implementation of an on‑site simulation programme during COVID‑19 and the assessment of its impact on medical students’ competence

**DOI:** 10.1007/s11845-022-03088-6

**Published:** 2022-06-28

**Authors:** Niall James McInerney, Mohammad Faraz Khan, Laoise Coady, Jeffrey Dalli, Maurice Stokes, Suzzane Donnelly, Helen Heneghan, Ronan Cahill

**Affiliations:** 1 UCD Centre for Precision Surgery, Mater Misericordiae, University Hospital, Eccles St, Dublin 7, Ireland; 2 UCD School of Medicine, Belfield, Dublin 4, Ireland; 3 St Vincent’s University Hospital, Elm Park, Dublin 4, Ireland

## Correction to: Irish Journal of Medical Science (2022) https://doi.org/10.1007/s11845-022-03057-z

Originally, the article was published with error. The author “Laiose Coady” should be correctly spelled as “Laoise Coady”.

The original article has been corrected.

